# Acute beetroot juice supplementation improves exercise tolerance and cycling efficiency in adults with obesity

**DOI:** 10.14814/phy2.14574

**Published:** 2020-10-14

**Authors:** Christian E. Behrens, Khandaker Ahmed, Karina Ricart, Braxton Linder, José Fernández, Brenda Bertrand, Rakesh P. Patel, Gordon Fisher

**Affiliations:** ^1^ Department of Nutrition Sciences The University of Alabama at Birmingham Birmingham AL USA; ^2^ Department of Pathology and Center for Free Radical Biology The University of Alabama at Birmingham Birmingham AL USA; ^3^ Department of Human Studies The University of Alabama at Birmingham Birmingham AL USA

**Keywords:** beetroot, exercise, nitrate, obesity

## Abstract

**Background:**

Exercise training improves health outcomes in individuals with obesity (IO); however, it remains challenging for IO to adhere to exercise. Thus, it is critical to identify novel strategies that improve exercise tolerance (ET) and adherence in IO. Beetroot juice (BRJ), high in inorganic dietary nitrate, consistently improves exercise performance in athletes, individuals with cardiopulmonary diseases, and nonobese lean individuals. These improvements may be explained by reduced oxygen uptake (VO_2_) during exercise, enhanced blood flow, and greater mitochondrial efficiency. To date, we are aware of no studies that have compared the effects of BRJ, sodium nitrate (NaNO3), and nitrate‐depleted BRJ (PLA) for improving ET and cardiometabolic health in IO.

**Purpose:**

Determine if BRJ improves ET, exercise efficiency (EE), and cardiometabolic health in IO and identify possible mechanisms of action.

**Methods:**

Vascular hemodynamic, submaximal‐ and maximal‐exercise VO_2_, and time to exhaustion (TTE) were assessed in 16 participants 2.5 hr following consumption of: 1) BRJ, 2) NaNO_3_, 3) PLA, or 4) CON.

**Results:**

A significant treatment effect was observed for submaximal exercise VO_2_ (*p* = .003), and TTE (*p* < .001). Post hoc analyses revealed lower VO_2_ during submaximal exercise in BRJ compared to PLA (*p* = .009) NaNO3 (*p* = .042) and CON (0.009), equating to an average improvement of ~ 7% with BRJ. TTE was greater for BRJ compared to other treatment arms, PLA (*p* = .008), NaNO3 (*p* = .038), and CON (*p*=<0.001), equating to ~ 15% improvement with BRJ. No significant changes were observed for other outcomes.

**Conclusions:**

Consumption of BRJ improved EE during submaximal exercise by 7%, and TTE by 15% compared to other conditions. These results suggest that BRJ may improve EE and exercise tolerance in IO.

## Graphical abstract

Exercise training improves health outcomes in individuals with obesity, however it remains challenging for individuals with obesity to adhere to exercise. Evidence suggests beetroot supplementation improves exercise‐related outcomes in athletes and the non‐obese lean, however the crossover of these effects onto adults with obesity is not clear. This study primarily aimed to determine if beetroot juice improves exercise tolerance and efficiency in individuals with obesity. Supplementation with beetroot juice significantly reduced oxygen uptake during submaximal exercise (~7%), and increased by ~9% during severe‐intensity exercise. Perhaps most notable was a ~22% improvement in time to exhaustion observed following beetroot juice intake compared to control. Taken together acute supplementation with BRJ may be effective for improving exercise tolerance and efficiency in individuals with obesity.

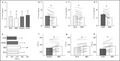

## 1 INTRODUCTION

Obesity is ranked among the top growing health concerns worldwide. In the United States alone, obesity affects over 93 million adults inflicting hundreds of billions of dollars in obesity‐related medical costs each year (Tremmel, Gerdtham, Nilsson, & Saha, 2017; Wolf & Colditz, 1998). This is especially concerning due to comorbidities associated with obesity such as hypertension, dyslipidemia, and type 2 diabetes. The successful treatment of obesity, particularly long term, has proven challenging, warranting continued investigation into alternative strategies that may reduce the incidence and severity of obesity and secondary cardiometabolic comorbidities.

Regular physical exercise has been shown to reduce the incidence of obesity and assist with weight maintenance following weight loss (Amisola & Jacobson, 2003; Ladabaum, Mannalithara, Myer, & Singh, 2014; McInnis, 2000; Shaw, Gennat, O'Rourke, & Del Mar, 2006). However, due to many of the biological consequences of obesity, individuals with obesity are often burdened by a diminished capacity and tolerance to exercise, often resulting in abstention and increased sedentarism (Bournat & Brown, 2010; Martínez‐González, Alfredo Martínez, Hu, Gibney, & Kearney, 1999; Tryon, Goldberg, & Morrison, 1992). One potential mechanism underlying reduced exercise tolerance is impaired nitric oxide (NO) production and bioactivity that manifests in decreased systemic oxygen delivery and/or skeletal muscle oxygen utilization (Lee‐Young et al., 2010; Pohl & Lamontagne, 1991). Obesity is associated with lower levels of NO production and bioavailability (Sansbury & Hill, 2014a, 2014b; Siervo, Jackson, & Bluck, 2011; Williams, Wheatcroft, Shah, & Kearney, 2002). Therefore, it is possible that strategies which increase NO production in individuals with obesity may improve oxygen delivery and/or utilization, increase exercise tolerance, and overall exercise participation, leading to improvements in secondary cardiometabolic comorbidities associated with obesity.

Previous work suggests supplementation with inorganic dietary nitrate (NO_3_^−^) found in many plant‐based sources such as beetroot, mustard leaf, spinach, and arugula, improves NO bioavailability and oxygen delivery and/or utilization leading to increased exercise tolerance and improvements in cardiometabolic health outcomes, such as blood pressure, endothelial function, and measures of inflammation and oxidative stress (Clements, Lee, & Bloomer, 2014; Clifford, Howatson, West, & Stevenson, 2015; Larsen et al., 2011; Lundberg & Govoni, 2004). Beetroot juice (BRJ), is exceptionally high in NO_3_^−^ and is commonly used to assess potential improvements in human health and exercise performance (Jones, Thompson, Wylie, & Vanhatalo, 2018). The mechanism by which BRJ leads to these improvements is that NO_3_^−^ is reduced to nitrite (NO_2_^−^) by oral bacterial nitrate reductase. Nitrite is then further reduced to NO in hypoxic and acidic compartments (Domínguez et al., 2017). The majority of these studies have focused on ergogenic benefits of dietary nitrate to trained athletes. Few clinical studies have investigated the effect on populations with chronic diseases, such as chronic obstructive pulmonary disease and heart failure with some reporting improvements in exercise and vascular outcomes, and others reporting no effect of treatment (Berry et al., 2015; Coggan et al., 2018; Eggebeen et al., 2016; Gilchrist et al., 2013). These studies have also provided insights into potential mechanisms underlying the ergogenic effects of BRJ including modulation of O_2_ metabolism characterized by faster pulmonary O_2_ consumption,( Breese et al., 2013). vasodilation increasing blood flow and oxygen delivery to muscles (Erzurum et al., 2007), improved ATP utilization by exercising myofibrils, and increased mitochondrial biogenesis (Larsen et al., 2011; Stamler & Meissner, 2001; Tong, Heim, & Wu, 2011). However, BRJ also contains many potent antioxidant compounds, which may confer additional benefits for exercise performance and cardiometabolic health. Thus, it is possible that BRJ can increase antioxidant capacity and blunt pro‐inflammatory pathways, providing a protective benefit against elevated oxidative stress observed in IO (Clifford et al., 2015; Georgiev et al., 2010; Kanner, Harel, & Granit, 2001; Vulić et al., 2014; Zielinska‐Przyjemska, Olejnik, Dobrowolska‐Zachwieja, & Grajek, 2009). It remains to be determined if the improvements in exercise and health outcomes following BRJ intake are due to increases in nitrate, antioxidant capacity, or a synergistic effect of both (Georgiev et al., 2010; Kanner et al., 2001; Reddy, Alexander‐ Lindo, & Nair, 2005).

To date, there have been very few clinical investigations into the efficacy of BRJ supplementation on obesity, although previous investigations have been similar in scope (Beals et al., 2017; Lara et al., 2015; Lima Bezerra et al., 2019; Rasica et al., 2018), to our knowledge there have been no clinical investigations specifically examining its effect on exercise tolerance in adults with obesity. Thus, the primary purpose of this study is to determine if BRJ supplementation improves exercise tolerance and markers of cardiometabolic health in individuals with obesity, and secondly to determine if the ergogenic and cardiometabolic health benefits of BRJ are due to increased nitric oxide bioavailability, increased antioxidant capacity, or a combination of these two. We utilize a three treatment crossover design involving a nitrate‐rich BRJ supplement containing all naturally occurring NO_3_^−^ and antioxidant properties, a nitrate‐depleted BRJ supplement containing only the naturally occurring antioxidant properties but negligible NO_3_^−^, and finally a nitrate‐matched sodium nitrate solution used as a surrogate vehicle for NO_3_^−^ delivery, without any added antioxidant compounds.

## 2 METHODS

### 2.1 Participants

The protocol for this study was reviewed and approved by the institutional review board at the University of Alabama at Birmingham (IRB‐300001210). Men and women (*N* = 16) were recruited for this randomized crossover study. Inclusion criteria were age 19 to 40 years, sedentary (not currently engaging in structured weekly physical activity), and obese (body mass index >30 kg/m^2^). Female participants were enrolled during the luteal phase of their menstrual cycle, lasting 12–14 days. Confirmation of menstrual cycle phase was self‐reported by female participants. Participants were nonsmokers and otherwise considered healthy with no history of chronic or degenerative disease. Participants were not currently taking any form of supplement or medication known to interfere with cardiometabolic or exercise measures associated with the study protocol. Participants were also asked to abstain from using antibacterial mouthwash (shown to interfere with NO_3_^−^ metabolism) and, antioxidant and pre/probiotic supplements for the duration of the experimental period.

### 2.2 Experimental design

**Figure 1:**
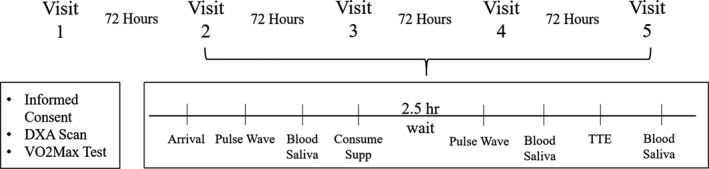
Study schematic

Participants visited the laboratory on five separate occasions over approximately an 18‐day period. Each visit was separated by at least a 72‐hr washout period to assure that effects of supplementation returned to baseline. On *visit 1* participants gave written informed consent to participate in the study. Total body composition was measured using dual‐energy X‐ray absorptiometry (DXA) and participants performed a graded incremental exercise test on an electronically braked cycle ergometer for the determination of VO_2peak_ and calculation of the gas exchange threshold (GET). *Visits 2–5* were identical in nature with the exception of the supplement consumed and consisted of blood and saliva collection at three separate time points, measurements of vascular health using pulse wave contour analysis (systolic/diastolic blood pressure, pulse rate, large, and small artery elasticity), and a time to exhaustion test (TTE). Study schematic detailed in Figure 1. Experimental visits all began at the same time of day (7:00 a.m.). Participants were asked to arrive to each experimental visit in a fasted state. All participants were instructed to maintain their regular diet throughout the course of their enrollment in the study. Diet recalls (24 hr) were obtained by a registered dietitian at each experimental visit. Data from 24‐hr recalls were entered into diet analysis software (NutriTiming® LLC, Atlanta, GA, USA) and examined for total calories, macronutrients, and other micronutrients known to influence antioxidant status. Dietary data were used to test for differences between and within participants for diet composition.

### 2.3 Supplementation

In a single‐blind crossover design, participants were randomized to one of four conditions at each visit: (a) NO_3_^−^ rich beetroot juice (BRJ; ~6.4 mmol of NO_3_^−^ per 70 ml; Beet it; James White Drinks, Ipswich, United Kingdom), (b) NO_3_^−^ depleted beetroot juice (PLA; ~0.04 mmol of NO_3_^−^ per 70 ml; Beet it; James White Drinks, Ipswich, United Kingdom), (c) sodium nitrate solution matched for the amount of NO_3_^−^ delivered by the BRJ (NaNO_3_; 0.5 grams of pure sodium nitrate, Sigma‐Aldrich *ReagentPlus*®, dissolved in 50ml of deionized water), or (d) no supplement control (CON). Although the BRJ and PLA conditions were indiscernible to participants, we acknowledge a limitation to this study design was our inability to further blind participants to the NaNO_3_ and CON conditions. At each visit, following baseline vascular measures and blood and saliva collection, participants were instructed to consume the supplement and then remained in the Clinical Research Unit in a seated and relaxed position for 2.5 hr.

### 2.4 Body composition measurement

Body mass was obtained in minimal clothing on a digital platform scale (Seca 514 mBCA, Seca North America, Chino, CA). Height was measured with shoes removed using a digital wall‐mounted stadiometer (Seca 264 Stadiometer, Seca North America, Chino, CA). Total body fat, lean mass, and visceral fat were measured by DXA with the use of a Lunar iDXA densitometer (GE Medical Systems, Madison, WI, enCORE Version 15). Participants were required to wear light clothing, remove all metal objects, and lie supine with arms at their sides during the scan.

### 2.5 Graded incremental exercise test

During *Visit 1* participants performed a graded incremental exercise test on an electronically braked cycle ergometer (Lode Excalibur Sport, Groningen, Netherlands) for the determination of VO_2peak_ and GET. Participants cycled for 3 min at 0 watts, after which the work rate was increased 30 watts per minute until the participant was unable to continue. Aerobic power was defined as the highest 30 s average volume of oxygen consumed before the participant's volitional exhaustion. Minute ventilation, oxygen uptake, and carbon dioxide production were continuously recorded by an open‐circuit spirometry system (TrueOne 2400, ParvoMedics, Salt Lake City, UT). Additionally, heart rate was continuously recorded (Polar T31 Coded Heartrate Transmitter, Polar Electro Oy, Kemple, Finland). VO_2peak_ confirmation was determined by meeting two of the following three criteria: (a) a plateau in oxygen consumption as workload increases, (b) a respiratory exchange ratio greater than 1.15, (c) a max heart rate within 10–12 beats or the age predicted heart rate max. Breath‐by‐breath VO_2_ (L/min) and VCO_2_ (L/min) was obtained and the participants GET was calculated using the V‐slope method (Schneider, Phillips, & Stoffolano, 1993).

### 2.6 Time to exhaustion test

During *Visits 2–5* participants underwent a TTE test 2.5 hr postsupplementation. Minute ventilation, oxygen uptake, and carbon dioxide production were continuously recorded by an open‐circuit spirometry system. Heart rate was also recorded throughout each test. Testing began with participants cycling for 3 min at a workload of 20 watts. After 3 min of warmup, the workload was increased to 90% of the workload performed at the participants GET for 5 min. After 5 min the workload was increased to 90% of the workload performed at the participants VO_2peak_ where cycling continued until volitional exhaustion or cycling cadence decreased below 60 rpm. The duration of exercise during this intensity was used to determine the participants TTE. Gas exchange data collected during the last 3 min of the second stage was used for submaximal VO_2_ comparison between arms and all gas exchange collected during the third stage of the test used for maximal VO_2_ comparisons.

### 2.7 Arterial elasticity test

Hemodynamic and arterial elasticity variables (Systolic/Diastolic blood pressure, mean arterial blood pressure, pulse rate, large/small artery elasticity total vascular impedance, systemic vascular resistance, cardiac output, estimated cardiac index) were obtained from the radial artery using a noninvasive calibrated pulse wave tonometer (HDI‐CR‐2000, Hypertension Diagnostics Inc. Eagen, MN). This analysis is based on a modified Windkessel model that allows evaluation of the large conduit arteries and the small microcirculatory arteries (Cohn et al., 1995). Data from Pulse wave tonometry were obtained at two time points in triplicate during each visit *(Visits 2–5)*. Upon arrival to the laboratory, participants were seated and stationary for at least 10 min prior to beginning the first pulse wave analysis. Following the first assessment, and baseline blood/saliva collection, participants received their randomized supplement and were asked to remain seated and stationary for 2.5 hr. Immediately following the 2.5 hr wait, a second pulse wave assessment was conducted. In short, in the seated position, a solid‐state pressure transducer array (tonometer) was placed over the participant's radial artery at the wrist and secured with a Velcro strap around the radial boney prominence. A wrist stabilizer was used to support the participant's arm to assure positioning and minimize movement and an automated oscillatory blood pressure cuff was placed on the contralateral arm. The waveform was calibrated by the oscillometric method. Once a stable measurement was achieved, a 30 s analog tracing of the radial waveform was digitized at 200 samples per second. BP measurements were taken before, during, and after the waveform assessment. The first maximum waveform recorded represents the work of the arteries after cardiac ejection and is representative of the large arteries, whereas the following rebound wave is reflective of small artery compliance. Total vascular impedance was determined from the modified Windkessel model evaluated at the frequency of the measured heart rate (Hales, 1733).

### 2.8 Blood and saliva collection

Blood and saliva samples were obtained at three time points during each experimental visit *(Visits 2–5)*. Samples were obtained at baseline (hour 0) with supplementation immediately following, 2.5 hr postsupplementation (hour 2.5), and immediately following TTE test (hour 3). Using 2.7ml sodium citrate, and 4.0ml EDTA vacutainers (Vacutainer, Becton Dickinson, Franklin Lakes, NJ), whole blood was obtained at each time point from an IV‐catheter placed in the antecubital vein. Within 2 min of collection samples obtained in sodium citrate tubes were centrifuged at 3,000 g for 2 min for subsequent nitrite and nitrate measurements. Samples obtained in EDTA tubes were centrifuged at 5,000 g for 10 min for subsequent antioxidant capacity measurements. Unstimulated saliva samples (~5ml) were collected in large 50ml conical tubes (Falcon) and subsequently separated into 1.5 ml Eppendorf tubes at each time point. Saliva samples were centrifuged at 3,000 g for ~5 min. Following centrifugation, plasma and saliva supernatant were separated into 500 μl aliquots, snap frozen in liquid nitrogen, and stored at −80°C for later determination of nitrite and nitrate concentration.

### 2.9 Total antioxidant capacity

Total plasma antioxidant potential was determined by the Ferric Reducing Ability of Plasma (FRAP) assay according to the methodology of Benzie and Strain (Benzie & Strain, 1996). Working FRAP solution was placed in a water bath and warmed to 37°C. Then, 10 μL of blank, samples, and ascorbate STDs were transferred by micropipette into designated well of 96‐well plate. Three hundred microliter of FRAP reagent was then added to all wells containing blank, samples, and standards. Ninety‐six‐well plate was then incubated for 4 min at 37°C before being read at 593 nm in a spectrophotometer (Molecular Devices – SpectraMax M3. Softmax Pro Version 6.3). Samples and standards were analyzed in triplicate, and FRAP values were expressed as vitamin C equivalents as determined by linear regression from a vitamin C curve (0–1000 μmol).

### 2.10 Measurement of nitrite and nitrate

After thawing, methanol was added to plasma and saliva (2:1 ratio), vortex mixed and supernatant collected. Nitrite and nitrate was then measured on methanolic extracts by HPLC‐coupled to the Griess reaction using the ENO‐20 (EiCom, Japan). Nitrate and nitrite levels were calculated by comparison to standard curves generated daily and adjusted for extraction efficiency (Joshipura, Munoz‐Torres, Morou ‐Bermudez, & Patel, 2017).

### 2.11 Statistical analysis

Sample size calculations for this study were based on mean changes and standard deviations for systolic blood pressure and volume of oxygen consumed (VO2) during submaximal exercise established from previous work in this area (Jajja et al., 2014; Jakeman & Maxwell, 1993). Given the mean changes and *SD* in SBP and submaximal VO2 following BRJ supplementation (*SD* = 5 mmHg and 0.10 LO2/min), a sample size of 11 for SBP and 6 for VO2 would be sufficient to yield a power of 80% to detect 20% differences with 95% confidence intervals. This study initially recruited 20 participants, with 16 participants completing all study requirements.

Participant characteristics from data collected during Visit 1 were analyzed using descriptive statistics, expressed as mean ± *SD*. Each continuous variable was checked for normality using Kolmogorov‐Smirnov tests. Normality was confirmed visually with QQ‐plot observations. For analyses assuming normally distributed data, log transformations were made for data not conforming to a normal distribution. Linear mixed models were used to determine main effects of treatment (BRJ, PLA, CON, and NaNO_3_) and time interaction (Visits 2–5) for all primary outcome variables (plasma and saliva NO_3_^−^/NO_2_^−^, FRAP, pulse wave variables, submaximal/maximal VO_2_, and TTE). Time and treatment were set as fixed effects for each of these models with treatment order set as a random effect. Studentized residuals falling outside of three standard deviations were considered outliers and removed from analysis; this resulted in two data points being excluded for saliva NO metabolites. Significant findings were determined by an alpha level of 0.05 for pairwise comparisons. Tukey's post hoc correction was used where significant effects were observed, however, given the nature of our small sample size and the possibility for overcorrection, we chose to report significance based on unadjusted pairwise comparisons. Relationships between NO metabolites and exercise outcomes were further explored using Pearson's correlation coefficients. Statistical tests were conducted using SAS Version 9.4 (Cary, NC).

## 3 RESULTS

Twenty participants were enrolled in this study. One participant was unable to perform exercise testing and 3 others were lost during follow‐up for reasons unknown, leaving 16 participants (11 male/5 female) who completed the study. Baseline characteristics of completed participants are presented in Table 1. There were no adverse health events reported or observed from supplementation or exercise testing.

**Table 1:** Participant characteristics (*n* = 16)

	Men (*n* = 11)	Women (*n* = 5)
Age	26 ± 6.1	30 ± 6.5
Height (cm)	179.3 ± 5.7	163.3 ± 6.5
DXA Total (kg)	114.1 ± 19.5	89.0 ± 11.7
DXA Lean (kg)	66.2 ± 7.6	44.0 ± 3.1
DXA Fat %	39.5 ± 5.6	48.6 ± 5.8
VO_2_Peak (mL/kg/min)	27.1 ± 3.7	25.5 ± 6.6
SBP (mmHg)	130 ± 14	107 ± 11
DBP (mmHg)	73 ± 8	60 ± 10

Note

Values are means ± *SD*.

**Figure 2:**
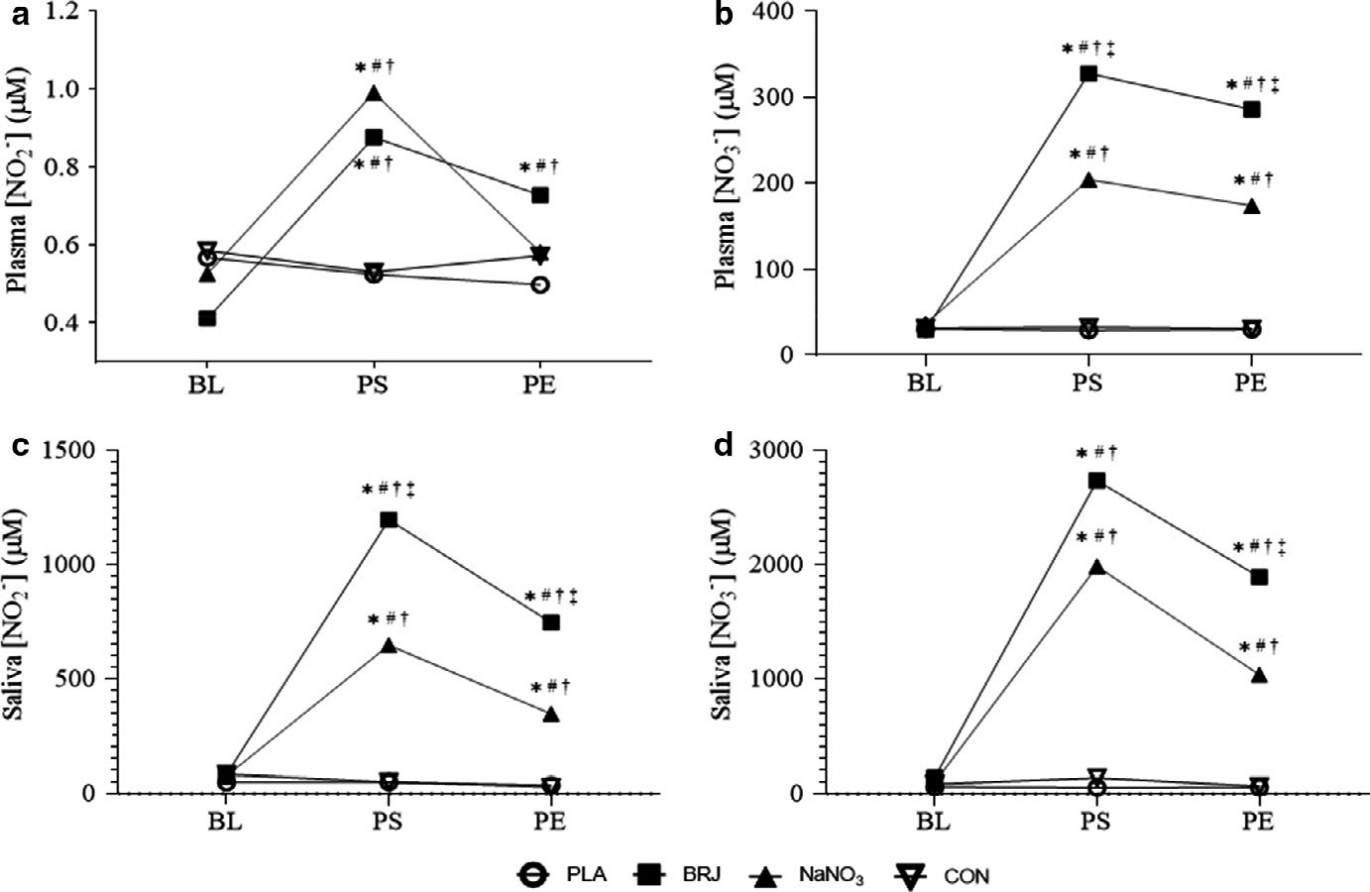
Changes in Plasma/Saliva [NO3‐/NO2‐]. (a and B) Plasma measures. (c and d) Saliva measures. Baseline (BL), 2 hr postsupplementation (PS), and postexercise (PE). *Significantly different from BL, #Significantly different from PLA, †Significantly different from CON, ‡Significantly different from NaNO3. (*p* < .05)

**Figure 3:**
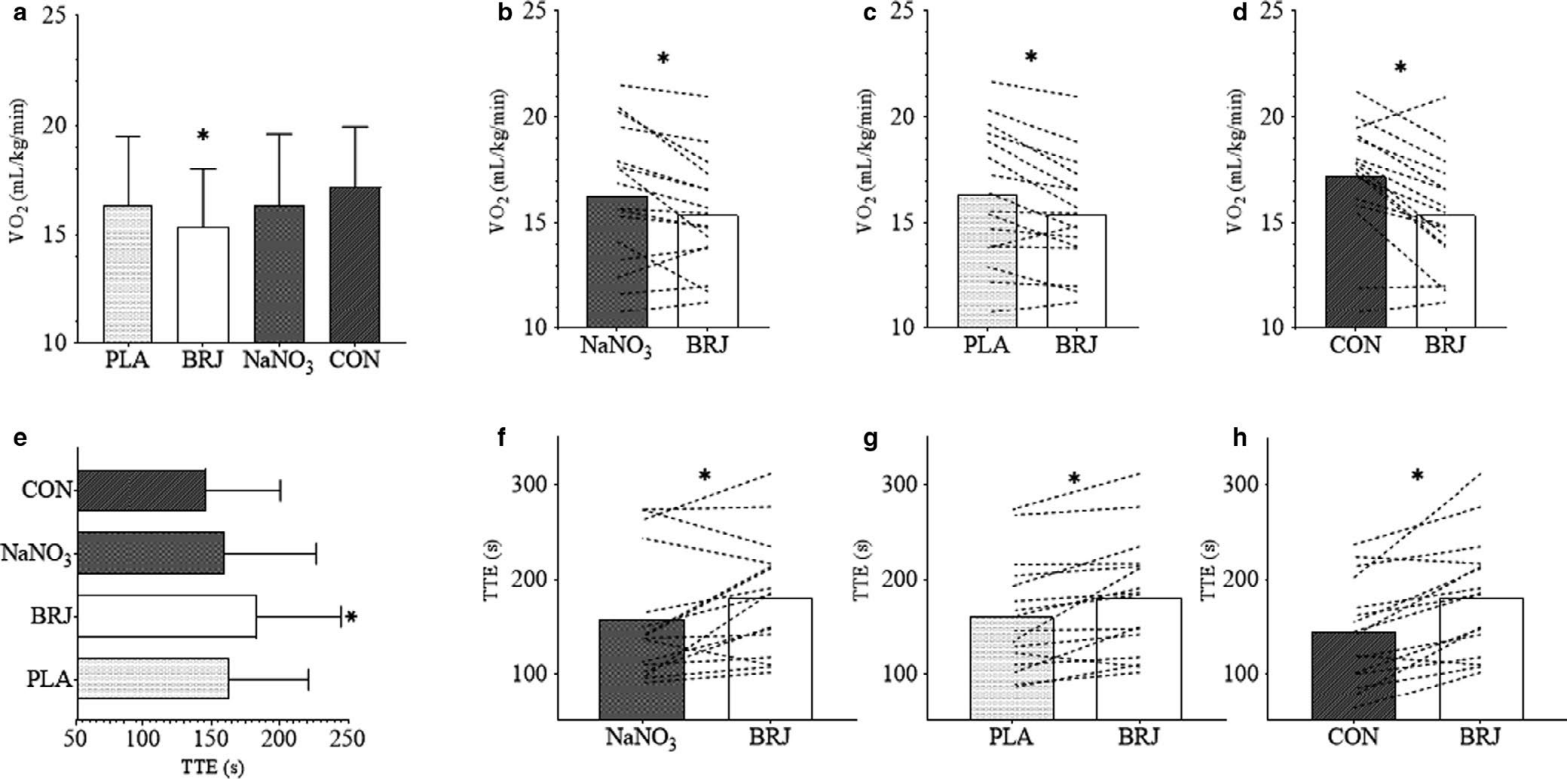
Exercise‐related Outcomes in Response to Treatment. (a) Mean VO_2_ response during submaximal exercise. (b‐d) Mean submaximal VO2 for BRJ compared to NaNO3, PLA, and CON, respectively. (e) Mean time to exhaustion (TTE). (f‐h) TTE for BRJ compared to NaNO3, PLA, and CON, respectively. *Significant difference between conditions. (*p* < .05)

**Figure 4:**
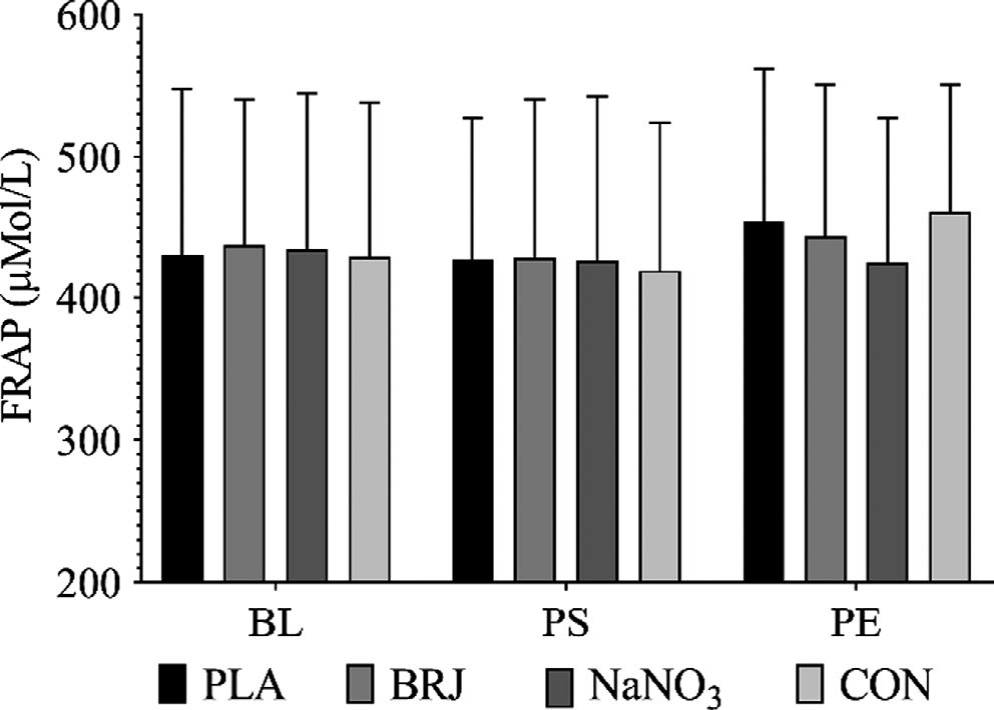
FRAP at baseline (BL), 2 hr postsupplementation (PS), and postexercise (PE). Values are expressed as µM ascorbate equivalents. No significant differences within or between treatments. (*p* < .05)

No significant differences were observed in diet composition within participants or across treatment arms (Table 2). Baseline saliva and plasma [NO_2_^−^] and [NO_3_^−^] were similar across participants and treatment arms. Absolute mean values and mean change in saliva and plasma [NO_2_^−^/NO_3_^−^] for each study arm are reported in Figure 2. Postsupplementation systolic and diastolic blood pressures were not different from baseline for any of the treatment arms (Table 3). Furthermore, there were no differences detected for other measures of vascular health (systolic/diastolic blood pressure, pulse rate, large and small artery elasticity) (Table 3). A significant main effect of treatment was observed for submaximal VO_2_ (*p* = .003) such that supplementation with BRJ resulted in a lower VO_2_ compared to PLA (*p* = .009), NaNO3 (*p* = .042), and CON (*p* = .009) (Figure 3), which reflects a 7% mean improvement in submaximal exercise efficiency. A significant effect for treatment was observed for TTE (*p* ≤ .001), with BRJ eliciting a significantly greater improvement in TTE compared to PLA (*p* = .008), NaNO_3_ (*p* = .038), and CON (*p* ≤ .001). A small but significant inverse association was observed for submaximal VO_2_ and postsupplementation plasma NO_2_^−^ (*p* = .04) depicted in Table 4. No significant changes were observed for total antioxidant capacity (Figure 4), or other exercise or vascular related measures.

**Table 2:** Participant diet composition

	PLA	BRJ	NaNO3	CON
Total Calories	2086 ± 892	2,527 ± 1,408	1883 ± 319	2,590 ± 846
Carbohydrate (g)	276 ± 170	300 ± 149	193 ± 61	263 ± 103
Protein (g)	90 ± 26	96 ± 64	94 ± 37	118 ± 38
Fat (g)	112 ± 63	116 ± 86	84 ± 20	122 ± 51
Vitamin A (IU)	391 ± 137	355 ± 112	369 ± 119	384 ± 127
Vitamin C (mg)	25 ± 11	28 ± 16	30 ± 19	31 ± 24
Vitamin E (mg)	3.1 ± 2.3	4.7 ± 4.1	3.3 ± 1.6	4.6 ± 2.8
Copper (mcg)	0.53 ± 0.40	0.93 ± 0.42	0.60 ± 0.39	0.80 ± 0.46
Manganese (mg)	1.9 ± 1.5	1.9 ± 1.2	1.8 ± 1.4	1.5 ± 0.90
Selenium (mcg)	66 ± 32	84 ± 77	68 ± 38	71 ± 33
Zinc (mg)	4.4 ± 2.9	4.5 ± 2.5	3.9 ± 1.9	4.7 ± 2.3

Note

Values are means ± *SD*.

**Table 3:** Blood pressure, Pulse rate, and Elasticity indices in response to supplementation (*n* = 16)

	PLA			BRJ			NaNO3			CON		
	Pre	Post	∆	Pre	Post	∆	Pre	Post	∆	Pre	Post	∆
SBP (mmHg)	121 ± 16	123 ± 14	2 ± 5	123 ± 15	121 ± 17	−2 ± 7	122 ± 17	122 ± 14	0 ± 6.5	124 ± 20	123 ± 17	−1 ± 12
DBP (mmHg)	68 ± 11	68 ± 9	0 ± 6	70 ± 11	69 ± 12	−1 ± 6	69 ± 10	69 ± 10	0 ± 4	70 ± 12	69 ± 11	−1.0 ± 5
PR (bpm)	69 ± 11	68 ± 11	−1 ± 6	71 ± 12	67 ± 11	−4 ± 4	71 ± 10	67 ± 9	−4 ± 5	70 ± 10	68 ± 10	−2 ± 5
LAE	16 0.0 ± 4.3	16.7 ± 5.2	0.7 ± 5.5	16.4 ± 3.2	16.2 ± 3.8	−0.2 ± 4.2	15.9 ± 4.1	16.8 ± 5.8	0.9 ± 6.3	16.0 ± 3.5	18.6 ± 6.6	2.6 ± 5.3
SAE	11.3 ± 4.5	10.7 ± 3.7	−0.6 ± 3.7	13.8 ± 13.8	10.6 ± 3.8	−3.2 ± 14.6	9.9 ± 3.3	10.9 ± 3.4	1.0 ± 3.3	11.9 ± 4.8	10.6 ± 3.1	−1.3 ± 5.1

Note

Values are means ± *SD*.

Abbreviations: LAE, large artery elasticity; SAE, small artery elasticity.

**Table 4:** Pearson Correlation Matrix for NO Metabolites and Exercise Outcomes

	PNO_2_^−^	PNO_3_^−^	SNO_2_^−^	SNO_3_^−^
Submax VO_2_	**−0.36**	−0.17	<0.01	−0.07
Maximal VO_2_	−0.11	0.11	0.19	0.18
TTE	0.051	0.09	0.17	0.14

Note

Bold values indicate *p* < .05.

P, plasma measures; S, saliva measures; TTE, time to exhaustion.

## 4 DISCUSSION

The main purpose of this study was to determine the effects of acute supplementation with inorganic dietary NO_3_^−^ in the form of BRJ, on exercise tolerance and cardiometabolic health among healthy men and women with obesity. Although recent studies have been similar in scope (Beals et al., 2017; Lara et al., 2015; Lima Bezerra et al., 2019; Rasica et al., 2018), to our knowledge, this is the first to report such findings in this population. The secondary purpose of this study was to explore the potential mechanisms by which these effects might occur by separating potentially salubrious components of BRJ (inorganic dietary nitrate and antioxidants) by treatment arm. In this study of 16 individuals with obesity we demonstrated that acute supplementation with inorganic dietary NO_3_^−^ rich BRJ increased plasma and saliva NO_3_^−^ and NO_2_^−^, decreased VO_2_ during submaximal exercise, and extended time to exhaustion. These results are reflective of increased submaximal exercise efficiency, and improved exercise tolerance. Despite previous investigations demonstrating improvements in blood pressure (Berry et al., 2015; Eggebeen et al., 2016; Jajja et al., 2014; Lima Bezerra et al., 2019; Webb et al., 2008), we did not find such improvements in our study.

We observed comparable results with previous studies in that supplementing with NaNO_3_ or NO_3_^−^ rich BRJ, increased both plasma and saliva NO_3_^−^/ NO_2_^−^ (Bailey, Fulford, et al., 1985; Bailey, Winyard, et al., 1985; Kelly, Vanhatalo, Wilkerson, Wylie, & Jones, 2013; Lansley, Winyard, Fulford, et al., 2011; Larsen, Weitzberg, Lundberg, & Ekblom, 2007). Our study, similar to others (Flueck, Bogdanova, Mettler, & Perret, 2016), further aimed to assess the difference in these responses between BRJ and a NaNO_3_ solution matched for nitrate content. Both arms resulted in a significant rise in plasma and saliva NO_3_^−^/ NO_2_^−^ from baseline. With the exception of plasma NO_2_^−^, the BRJ treatment elicited a significantly greater response compared to NaNO_3_. These results suggest that other components in concentrated BRJ enhance nitrate metabolism (absorption/transport) into the blood and saliva and support data presented by Jonvik et al who demonstrated that acute supplementation with nitrate‐matched beetroot, rocket salad, and spinach beverages increased plasma NO_3_^−^ and NO_2_^−^ to a greater extent than NaNO_3_ (Jonvik et al., 2016). BRJ contains the amino acid betaine, and polyphenols quercetin and resveratrol. Although there were no differences detected in antioxidant capacity from baseline to postsupplementation across study arms, it is possible there may be synergistic interactions between dietary NO_3_^−^ and these compounds that lead to improved ergogenic effects. Notably, the antioxidant compounds in BRJ have been linked to improvements in mitochondrial efficiency, increased aerobic capacity, improvements in endurance, strength, and power (Alway et al., 2017; Davis, Murphy, Carmichael, & Davis, 2009; Hoffman, Ratamess, Kang, Rashti, & Faigenbaum, 2009). This idea is supported by results from the present study showing greater improvement in exercise‐related outcomes following BRJ consumption compared to NaNO_3_ despite the putative endpoint, plasma NO_2_^−^ being similar. Indeed, this suggests the involvement of other factors which may contribute to these NO mediated outcomes. Previous studies assessing the effectiveness of exogenous antioxidant supplementation alone are widely inconclusive, with some suggesting improvement in performance and/or physiological markers associated with exercise (Clarkson & Thompson, 2000; Jakeman & Maxwell, 1993), and others reporting no effect (Gey, Cooper, & Bottenberg, 1970). Differences in training status, affecting endogenous antioxidant production, diet, along with time, type, and intensity of exercise has all been cited as possible reasons for the inconsistencies observed with previous antioxidant‐alone investigations. Antioxidant compounds in BRJ (vitamin C and polyphenols) have been shown to facilitate NO_2_^−^ reduction to NO in the gut (Peri et al., 2005; Rocha, Gago, Barbosa, & Laranjinha, 2009). A study by Ogawa *et al* found that the generation of NO from reduced NO_2_^−^ was enhanced by high‐phenolic beverages (Ogawa & Mochizuki, 2013). Therefore, the differential response may rise from more efficient conversion of NO_2_^−^ to NO, catalyzed by phenolic compounds, vitamin C, and other antioxidants within BRJ. An interesting area of future research would be elucidating the independent and/or synergistic contributions these compounds may elicit, and further determine the dose/duration of supplementation necessary to detect optimal effects.

The majority of previous investigations have focused on the ergogenic potential of BRJ in athletic and healthy individuals, with many, but not all demonstrating improvement in performance time, submaximal O_2_ consumption, mitochondrial efficiency, and blood pressure (Cermak, Gibala, & van Loon, 2012; Domínguez et al., 2017; Lansley, Winyard, Bailey, et al., 2011; Larsen et al., 2007). Notably, effects of dietary nitrate are inconsistently observed in some disease settings such as type 2 diabetes (Beals et al., 2017; Gilchrist et al., 2013) underscoring the need to assess whether this pathway is functional in disease. Many individuals with obesity present with a reduced ability to participate in physical exercise characterized by reduced cardiorespiratory fitness and exercise intolerance (Salvadori et al., 1999; Tryon et al., 1992). Similar to results with healthy volunteer (Bailey, Fulford, et al., 1985; Bailey, Winyard, et al., 1985; Lansley, Winyard, Fulford, et al., 2011; Larsen et al., 2007), we demonstrated that acute supplementation with BRJ improved VO_2_ during submaximal cycling by ~ 7% over all other experimental arms in adults with obesity. It has been suggested that these improvements could in part be due to increased circulating NO_2_^−^ which through a series of different mechanisms can undergo a one‐electron reduction to NO (Lundberg & Weitzberg, 2009) This NO_2_^−^ to NO reduction is partially regulated by declines in O_2_ tension and pH which mirrors increases in exercise intensity (Case y, Treichler, Ganger, Schneider, & Ueda, 2015; Gaebelein & Ladd, 1986) A study by Bailey *et al* attributed the reduced O_2_ cost of submaximal, moderate‐intensity exercise to a reduction in ATP cost of force production (Bailey, Fulford, et al., 1985) This was further capitulated by Larsen *et al* showing lower O_2_ demand during submaximal work in healthy trained men (Larsen et al., 2007). Other potential mechanisms include improvements in mitochondrial and Ca^2+^ handling proteins that may improve metabolic and contractile function (Larsen et al., 2011). Obesity is associated with a higher O_2_ cost for submaximal exercise (Salvadori et al., 1999) and reduced VO_2peak_ (Green, O'Connor, Kiely, O'Shea, & Egana, 2018) which may require increased work and metabolic stress when participating in activities of daily living. The results from this study and others, suggest that acute NO_3_^−^ supplementation decreases the cost of submaximal work. Future studies should more closely examine the effects of both acute and chronic supplementation on functional capacity during lower intensity activities of daily living in individuals with obesity, perhaps improving functional outcomes and quality of life.

We found that BRJ improved exercise tolerance during high‐intensity cycling by ~15% over all other treatment arms with as high as a 22% improvement when compared to CON. In addition to the aforementioned reasons, improvements in high‐intensity exercise following BRJ supplementation is consistently attributed to alterations in VO_2_ kinetics, affecting finite substrate utilization and availability during exercise (i.e., rate of PCr breakdown and ATP generation from anaerobic glycolysis—metabolites of which are implicated in skeletal muscle fatigue) (Bailey, Varnham, et al., 1985; Breese et al., 2013; Murgatroyd, Ferguson, Ward, Whipp, & Rossiter, 1985). Although we did not measure VO_2_ kinetics in this study, Bailey et al and others have shown that BRJ supplementation speeds phase II VO_2_ kinetics during high‐intensity exercise, possibly linked to enhanced muscle vasodilation, blood flow, and O_2_ supply (Bailey, Varnham, et al., 1985). This is perhaps most important in type II muscle fibers where O_2_ delivery often lags behind O_2_ demand ultimately leading to oxidative inefficiency and fatigue during severe‐intensity exercise (Grassi, Rossiter, & Zoladz, 2015; McDonough, Behnke, Padilla, Musch, & Poole, 2005). Indeed, a similar study by Rasica *et al* illustrated a reduced rate of VO_2_ increase during severe‐intensity exercise with BRJ supplementation, in adolescents with obesity, suggesting improved efficiency (Rasica et al., 2018). The observed improvement in exercise tolerance with BRJ during high‐intensity exercise is noteworthy considering that high‐intensity interval training has been shown to be a highly effective intervention for improvement in weight and health status in individuals with obesity (Fisher et al., 2015; Gillen et al., 2016).

Strengths of this study include its design, utilizing a partially blinded, four condition, crossover design including a BRJ supplement, a placebo supplement with negligible NO_3_^−^ content, a NaNO_3_ solution matched for BRJ NO_3_^−^ content, and a no treatment control. Many previous investigations have speculated other bioactive compounds within BRJ may act synergistically with NO_3_^−^. By including a NO_3_^−^ matched NaNO_3_ treatment in our study, we were able to examine potentially mechanistic differences in response between BRJ, PLA, and NaNO_3_ with our results indeed suggesting the presence of a synergistic effect from BRJ. Weaknesses in our study include the inability to blind participants to treatment (NaNO_3_ treatment and CON), a small sample size, and racial homogeneity, making it difficult to generalize our findings. However, only 2 of our 16 participants failed to significantly improve their TTE when supplemented with BRJ compared to CON.

In conclusion, in individuals with obesity, acute supplementation with inorganic dietary nitrate‐rich BRJ, significantly increased [NO_3_^−^] and [NO_2_^−^] measured in both saliva and plasma. Additionally, VO_2_ during submaximal exercise was decreased by ~7%, and perhaps most notable was a ~22% improvement in time to exhaustion observed in the BRJ condition over control. These results taken together suggest that acute supplementation with BRJ is an effective strategy for improving exercise tolerance and efficiency in individuals with obesity. With reduced participation in physical exercise being hallmark of those with obesity, perhaps in part due to insufficient or impaired NO bioavailability, regular supplementation with BRJ may improve NO status and in turn improve exercise‐related outcomes in individuals with obesity. Regular physical exercise has been implicated in the long‐term success of weight loss maintenance. Thus, identifying novel strategies to improve exercise participation among individuals with obesity is of critical importance. Outcomes from this study suggest that BRJ, may be an easily obtained, cost‐effective nutraceutical to assist easing the burden of exercise and perhaps improve participation in and adherence to exercise.

## CONFLICT OF INTEREST

The authors have no conflict of interest.

## AUTHOR CONTRIBUTION

GF, CB, RP, KR, BL, and KA performed the experiments, GF, CB, KA, KR, and JF analyzed data and interpreted results of experiments. CB drafted manuscript. GF, RP, and JF edited and revised manuscript. GF, CB, and RP conceptualized and designed the experiments. All authors approved final version of manuscript.

## ETHICAL STATEMENT

The protocol for this study was reviewed and approved by the institutional review board at the University of Alabama at Birmingham. Informed consent was obtained from all study participants.
